# Quality of life of Brazilian industrial workers: a review article

**DOI:** 10.47626/1679-4435-2020-562

**Published:** 2020-12-11

**Authors:** Thais Jorrana de Oliveira Santos, Cristiane Estevão Tavares, Fabiana Pavan Viana, Rayne Ramos Fagundes

**Affiliations:** 1 Programa de Graduação em Fisioterapia, Pontifícia Universidade Católica de Goiás (PUC Goiás) - Goiânia (GO), Brazil; 2 Programa de Graduação em Fisioterapia, Universidade Estadual de São Paulo - São Paulo (SP), Brazil; 3 Programa de Graduação em Direito, Pontifícia Universidade Católica de Goiás (PUC Goiás) - Goiânia (GO), Brazil; 4 Programa de Pós-Graduação em Fisioterapia, Universidade Federal de São Carlos - São Carlos (SC), Brazil; 5 Escola de Ciências Sociais e da Saúde, Pontifícia Universidade Católica de Goiás (PUC Goiás) - Goiânia (GO), Brazil; 6 Programa de Graduação em Fisioterapia, Universidade Estadual de Goiás - Goiânia (GO), Brazil; 7 Programa de Pós-Graduação em Ciências da Saúde, Universidade Federal de Goiás (UFG) - Goiânia (GO), Brazil

**Keywords:** workers, quality of life, industry

## Abstract

Workers in general have their quality of life affected; however, in the industrial setting, they are exposed to poor and unhealthy conditions in their workplaces and to different occupational hazards depending on their role, sector of activity, and climatic conditions. This study aimed to analyze the quality of life of Brazilian industrial workers. This is an integrative review. The PICo strategy was used to prepare the research question, and each letter represents one component of the question - population (P), workers; interest (I), quality of life; and context (Co), Brazilian industry. The search and selection process was conducted in the Scientific Electronic Library Online (SciELO) and PubMed databases. The search was conducted from August to September 2019, and studies published in the past 10 years were selected. In most workers, work-related QL deficits were identified mainly in the domains of general health and vitality in the Medical Outcomes Study 36-Item Short-Form Health Survey (SF-36) and of social relationships and environment in the World Health Organization Quality of Life short version (WHOQOL-Bref). The factors that represented risks for quality of life included exposure to noise in the workplace, repetitive strain injury/work-related musculoskeletal disorders, occupational low back pain, occupational stress, and work-related fatigue. Few studies assessing quality of life of industrial workers were found. The reviewed studies showed that quality of life deficits are related to vitality, physical functioning, general health, environment, and psychological health of these workers.

## INTRODUCTION

The World Health Organization (WHO)^1^ understands quality of life (QoL) as the perception that individuals have about their position in life in the context of the culture and value systems in which they live and in relation to their goals, expectations, standards, and concerns. It is a broad concept affected in a complex way by each person’s physical health, psychological status, personal beliefs, social relationships, and relationship with important characteristics of the environment.^2^

Workers in general have their QoL affected. However, in the industrial setting, they are exposed to poor and unhealthy conditions in their workplaces and to different occupational hazards depending on their role, sector of activity, and climatic conditions. These hazards may be physical, chemical, ergonomic, or of accident. Work is often exhausting, stressful, unhealthy, inhumane, and with strict schedules, and it may cause irreversible damage to workers’ health,^3^^,^^4^ thereby affecting their QoL.

To achieve a good QoL, individuals must balance the conditions inside and outside the workplace, consisting of physical, psychological, and social factors. Thus, the workplace and the reactions generated by it in the lives of workers must be accounted for.^5^

In view of those observations, developing research on workers’ QoL is important, as it makes companies aware that investing in QoL is a means of contributing to a growth in society and the global economy. After all, most people spend most of their time at work,^6^ and the implementation of strategies to improve workers’ QoL leads to better performance and productivity.^7^

Most studies on workers’ health address other issues, such as hearing health, psychosocial disadvantages, repetitive strain injury/work-related musculoskeletal disorders (RSI/WMSDs) and occupational low back pain,^8^ mental disorders, occupational stress, and exposure to noise.^9^^,^^10^ There are few articles addressing that group’s QoL. Thus, the present study aimed to assess the QoL of Brazilian industrial workers.

## METHODS

An integrative review was conducted. This method allows developing relevant research that supports decision-making and clinical practice improvement, contributing to a greater knowledge of some researched topics, in addition to showing gaps that need to be filled in further studies. The general purpose of a literature review is to gather knowledge on a specific topic, helping to conduct a meaningful study that is crucial for the researchers.^11^

The PICo strategy was used to prepare the research question, and each letter represents 1 component of the question: population (P), interest (I), context (Co). The PICo strategy of this study was the following: P, workers; I, QoL; and Co, Brazilian industry. The search and selection process was conducted in the Scientific Electronic Library Online (SciELO) and PubMed databases. The search was conducted from August to September 2019, and studies published in the past 10 years were selected.

The following controlled (co) and noncontrolled (nco) descriptors were used to search for articles: trabalhadores/workers (co) OR industriários/ industrialists (nco) AND qualidade de vida/quality of life/life quality (co) AND indústrias/industry (co) OR indústrias brasileiras/Brazilian industry (nco). The inclusion criteria defined for article selection were the following: articles written in Portuguese, English, or Spanish that included the combinations of those descriptors; full-text articles that addressed the topic; and articles of cross-sectional, longitudinal, prospective, and retrospective studies published and indexed in these databases in the past 10 years. Studies of retired industry workers, studies of workers from other sectors or from non-Brazilian industries, studies that did not administer QoL questionnaires, studies that evaluated the effects of physical therapist treatment or intervention, as well as literature reviews, theses, monographs, and dissertations, were excluded.

Two tests were used for article selection: relevance test 1 (administered to titles and abstracts of articles) and relevance test 2 (administered to full-text articles) (Chart 1).

**Chart 1:** Relevance tests 1 and 2.

Relevance test 1 administration form		
** Inclusion criteria**	**Yes**	**No**
Are Brazilian industrial workers used as the study sample?		
Was the article published between 2010 and 2019?		
Is the article written in Portuguese, English, or Spanish?		
Is the article available in full-text?		
** Exclusion criteria**	**Yes**	**No**
Is it a literature review, thesis, or dissertation article?		
Is it a study that evaluated the effects of physical therapist treatment or intervention?		
Did the study use any quality of life assessment questionnaire?		
Did the study include workers from other sectors or from non-Brazilian industries?		
Did the study include retired industrial workers?		
**Relevance test 2 administration form**		
** Inclusion criteria**	**Yes**	**No**
Is quality of life of Brazilian industrial workers the focus of the study?		
Does the study describe quality of life?		
** Exclusion criteria**	**Yes**	**No**
Is it focused on other aspects of the lives of industrial workers?		
Did the study include workers from other countries?		

After reviewing and analyzing the articles, data on industrial workers’ QoL were tabulated, and the results were presented and described.

## RESULTS

**Figure 1:**
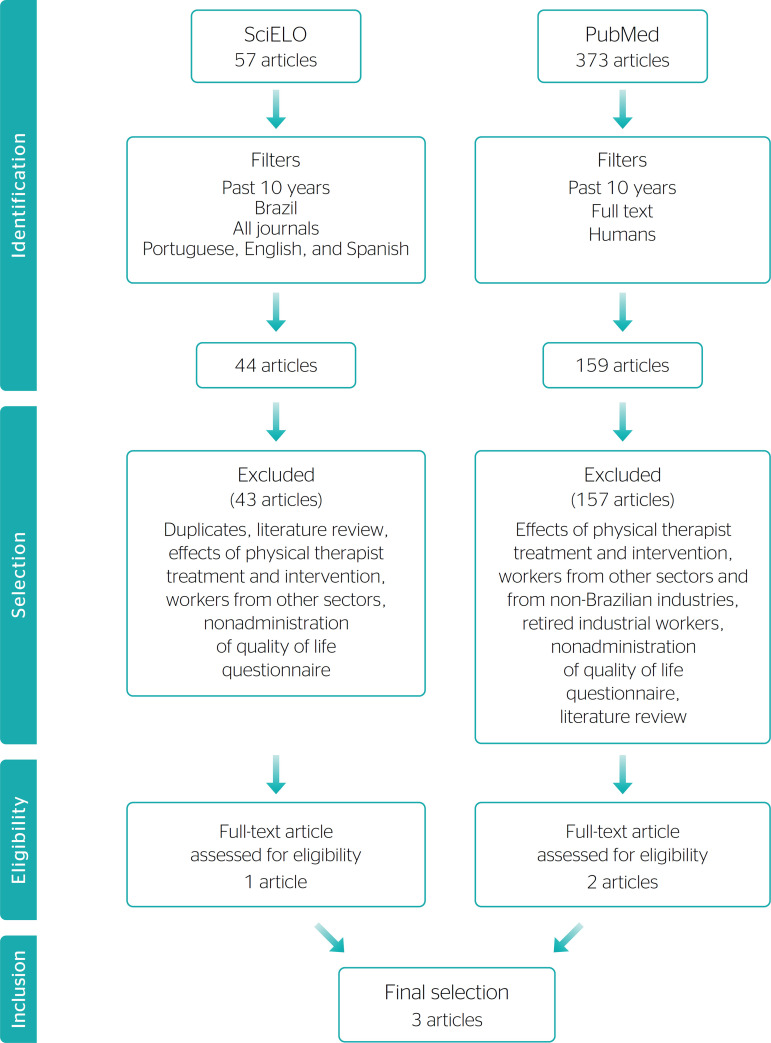
Flow diagram of sample selection.

The search and selection process for articles in the databases yielded only 3 scientific papers^12^^-^^14^ that met the study objectives and inclusion and exclusion criteria (Figure 1). Regarding the main results of the reviewed studies, a deficit in work-related QoL was identified in most workers. The factors that represented risks to QoL included exposure to noise in the workplace, RSI/ WMSDs, occupational low back pain, occupational stress, and work-related fatigue.

Table 1 presents a synthesis of the workers’ QoLrelated articles that were reviewed, and Tables 2 and 3 show quantitative QoL results according to the administered questionnaire. The studies were described in terms of author(s), title, year, setting, language, objectives, study design/sample profile/instruments, and results.

**Table 1:** Description of the reviewed articles on quality of life (QoL).

Author(s)	Title	Year/setting/language	Objective	Study design/sample profile/instruments	Results
Costa et al.^13^	Capacidade para o trabalho e qualidade de vida de trabalhadores industriais [Work ability and quality of life of Brazilian industrial workers]	2012, Brazil, Portuguese	To evaluate and compare QoL and work ability of industrial workers by means of two specific instruments	The study recruited 100 workers, 34 men and 66 women, with a mean age of 34.8 years. The sample consisted of night shift workers from the production department of a medium-sized company in the state of São Paulo that produces school and office supplies. The WHOQOL-Bref questionnaire was used to assess QoL.	The mean QoL score was 66.52, on a scale of 0-100. The WHOQOL-Bref scores were 69.40 for physical health, 68.91 for psychological health, 71.96 for social relationships, and 55.79 for environment.
Pimenta et al.^14^	Qualidade de vida e excesso de peso em trabalhadores em turnos alternantes [Quality of life and overweight in alternating shift workers]	2019, Brazil, Portuguese	To identify the perception that alternating shift workers from a mining company in the region of Inconfidentes, Minas Gerais, Brazil, have about their QoL and to analyze its association with indicators of excess body adiposity	This cross-sectional study recruited 437 workers on alternating shifts, working 6 hours per shift and having 12 hours to rest between shifts, with at least 1 cardiovascular risk factor. The SF-36 questionnaire was used to assess QoL.	The scores for the different QoL domains ranged from 67 to 100; the general health and vitality domains had the lowest scores, with median scores of 67 and 80, respectively, and increased body fat had a negative correlation with the general health, vitality, and physical functioning domains. No association was found between QoL scores and the number of working hours in the alternating shift scheme.
Carvalho Junior et al.^12^	Avaliação da qualidade de vida relacionada à saúde de cortadores de cana-de-açúcar nos períodos de entressafra e safra [Assessment of health-related quality of life of sugarcane cutters in the pre-harvest and harvest periods]	2012, Brazil, Portuguese	To assess healthrelated QoL in sugarcane cutters from the sugar-alcohol agroindustry	This longitudinal study evaluated 44 sugarcane cutters, both smokers and nonsmokers, at 3 time points: preharvest, end of the third month of harvest (midharvest), and late harvest. Health-related QoL was assessed by the SF-36 questionnaire.	Twenty-seven percent of workers were smokers. At the end of preharvest, 23% of workers dropped out. There was a significant decrease in the vitality domain at late harvest compared to preharvest. Dropouts had a higher score in the social functioning domain compared to the group that remained working. Nondropouts were divided into 2 groups: nonsmokers and smokers. A higher percentage of positive respondents was observed among nonsmokers in the physical role, emotional role, and social functioning domains in the 3 months of harvest and in the general health and social functioning domains in the 6 months of harvest when compared to smokers.

WHOQOL-Bref: World Health Organization Quality of Life-Bref; SF-36: Medical Outcomes Study 36-Item Short-Form Health Survey.

**Table 2:** Description of the results of the reviewed article according to World Health Organization Quality of Life-Bref (WHOQOLBref) quality of life scores.

Questionnaire	Physical health	Psychological health	Environment	Social relationships	Overall mean
WHOQOL-Bref	69.40	68.91	55.79	71.96	66.52

**Table 3:** Description of the results of the reviewed articles according to the Medical Outcomes Study 36-Item Short-Form Health Survey (SF-36) quality of life scores.

Questionnaire	Time point	PF Median (25-75%)	RP Median (25-75%)	BP Median (25-75%)	GH Median (25-75%)	VT Median (25-75%)	SF Median (25-75%)	RE Median (25-75%)	MH Median (25-75%)
**SF-36**		95 (90 -100)	100 (100-100)	84 (72-100)	67 (57-77)	80 (70-90)	100 (87.5-100)	100 (100-100)	88 (80-96)
**SF-36**	Preharvest	100 (90-100)	100 (75-100)	100 (72-100)	81 (68-92)	87.5 (70-95)	100 (78-100)	100 (66-100)	82 (76-92)
	Midharvest	100 (100-100)	100 (100-100)	100 (72-100)	82 (68-92)	82.5 (70-100)	100 (75-100)	100 (100-100)	86 (73-96)
	Late harvest	100 (100-100)	100 (100-100)	84 (62-100)	77 (59-92)	70 (61-83)	100 (75-100)	100 (100-100)	84 (72-88)

BP: bodily pain; GH: general health; MH: mental health; PF: physical functioning; RE: role-emotional; RP: role-physical; SF: social functioning; VT: vitality.

Regarding the methods that were applied, the first study used a cross-sectional approach, the second was longitudinal, and the third does not mention the study design. As for the research setting, 2 studies were conducted in the state of São Paulo and 1 in the state of Minas Gerais. All studies were published from 2010 to 2019.

Figure 1 describes the different stages of the review that yielded the selection of the 3 articles.

## DISCUSSION

The search strategy used in this review identified 3 studies that met all inclusion criteria. Two studies used the Medical Outcomes Study 36-Item Short-Form Health Survey (SF-36) questionnaire to assess work-related QoL, and one study used the World Health Organization Quality of Life short version (WHOQOL-Bref) questionnaire to characterize QoL. The domains with lowest scores were environment and psychological health for WHOQOL-Bref and general health and vitality for SF-36.

The SF-36 Portuguese version consists of 36 items covering 8 domains: physical functioning, physical role functioning, bodily pain, general health, vitality, social functioning, emotional role functioning, and mental health. Each domain is assigned a score ranging from 0 to 100; the higher the score, the better the perceived QoL. The questionnaire is self-administered, and researchers are only required to observe whether any questions were not answered, which causes invalidation. The SF-36 scores are calculated using the following steps: calculation for each domain and sum of the scores obtained in each item relative to the corresponding domain.^15^^,^^16^

The WHOQOL-Bref consists of 26 questions grouped into 4 domains: physical health, psychological health, social relationships, and environment. Question 1 refers to overall QoL and question 2 to satisfaction with health. Then there are 24 questions divided into the physical health, psychological health, social relationships, and environment domains.^17^ Thus, this is an instrument that can be used both for healthy populations and for populations affected by medical conditions and chronic diseases.^18^^,^^19^ In addition to being cross-cultural, the WHOQOL assessment instruments value the individual perception of QoL in different groups and situations.^20^ The Portuguese version was produced according to the methods recommended by the WHOQOL center for Brazil and presented satisfactory psychometric characteristics.^14^

In the study conducted by Costa et al.,^13^ which used the WHOQOL-Bref to assess QoL of night shift workers in a manufacturer of school and office supplies, the social relationships and environment domains showed significantly lower mean scores for women. Female QoL is commonly related to women’s performance as workers, wives, mothers, and housewives. Therefore, nonoccupational activities such as housework and child care create extra working hours.^13^^,^^21^ These factors may have contributed to explain lower female scores in perceived QoL. Such circumstance, associated with other risk factors and little time to relax or perform some physical activity, may contribute to a low perceived QoL.^22^ There is also a higher frequency of diseases that affect women more frequently, such as psychiatric disorders not associated with psychoactive substance or alcohol abuse^23^ and musculoskeletal diseases.

In the study conducted by Carvalho Junior et al.,^12^ health-related QoL in sugarcane cutters from the sugar-alcohol agroindustry, as well as in workers who are specifically within the industrial environment, was affected as both groups are exposed to working conditions that require poor posture, causing joint overload, and activities that involve excess muscular strength, associated with mechanical activation of industrial equipment and use of working tools.^24^ They are also subjected to occupational stress, pressure for productivity, unhealthy environments, and excess workload. With regard to sugarcane cutters, they were evaluated at 3 time points: preharvest, the period when they plant sugarcane; midharvest, 3 months after the beginning of the period in which they cut sugarcane that was previously burned; and late harvest. At preharvest, the lowest QoL scores of these workers were in the general health domain; at midharvest and late harvest, the lowest scores were in the vitality domain, probably because, over this long period of work, workers gradually showed decreased energy level and increased fatigue due to an intense workload.^12^

The fact that at preharvest workers experienced less fatigue than at harvest may be related to the correlation between workload and fatigue, previously described by Yamazaki et al.^25^ According to these authors, when a worker changes to a role that requires more intense activity, the level of fatigue increases. This result was also similar to that found in the study conducted by Carvalho Junior et al.,^12^ as harvest is extremely intense and more exhausting than preharvest. Fatigue in the workplace has also been addressed in studies of workers of different roles, such as small employers, traders, sewers, administrative agents, and police officers, in which fatigue was found to tend to be particularly associated with unstable employment, low control of work , low social status, low income, increased working hours, reduced rest periods, and full-time work.^8^^,^^26^^-^^28^

At the end of preharvest, 23% of workers dropped out, which can be explained by their health status in the period when sugarcane was planted. This may also suggest poor working conditions and insufficient medical care for this population, which is consistent with a study conducted by Alves,^29^ which collected data from the Migrant Pastoral Service of Guariba, state of São Paulo, and reported that, in the periods between harvests 2004/2005 and 2006/2007, 14 sugarcane cutters died in the sugarcane-producing region of São Paulo. They were mostly young workers, aged 24 to 50 years, migrants from other parts of the country (northern Minas Gerais, Bahia, Maranhão, and Piauí), and some of them were found to have vague causes of death, including cardiac arrest, respiratory failure, and stroke. Friends and families reported that, before dying, they had complained of overwork, body aches, cramps, shortness of breath, fainting, etc.

There was a significant decrease in the vitality domain at late harvest compared to preharvest. This shows that workers had decreased energy level and increased fatigue due to an intense workload. Dropouts had a higher score in the social functioning domain compared to nondropouts. The relationship between QoL in social functioning and dropping out of work or not can be explained by the fact that stressful conditions in the workplace may cause damage to the physical and mental health of workers, and satisfaction with work may be related to remaining in or abandoning their roles.^12^^,^^30^^,^^31^

In the studies that used SF-36, the general health and vitality domains were those with lowest scores, which may be related to decreased energy levels, increased fatigue, intense workload, poor health and working conditions, insufficient medical care, low self-esteem, and increased body adiposity.^12^^,^^14^ These results are similar to those of the study conducted by Pimenta et al.^14^ to evaluate workers from a mining company, in which the general health and vitality domains had the lowest scores, with median scores of 67 and 80, respectively.

In the studies that used WHOQOL-Bref, the environment and psychological health domains had the lowest scores. With regard to the environment domain, low scores may be related to noise in the workplace, poor working conditions, poor posture at work, tinnitus, muscle pain, and headache. A low score in the psychological health domain may be related to emotional problems deriving from low self-esteem, interaction between family and professional life, extra working hours including housework and activities involving child care, and work shifts, which may cause difficulties in family life and restrictions on social life and leisure activities with a partner, friends, and children.^13^^,^^32^^-^^34^

In a study conducted by Gomes et al.,^21^ which compared the work of home sewers with that of workshop sewers in Cianorte, state of Paraná, the lowest scores were usually in the bodily pain, general health, vitality, and mental health domains. In that study, although the industry (workshop) environment exposed workers to several factors that affected their QoL, a more severely compromised QoL was identified in home sewers compared to workshop sewers.

The scores of home sewers were significantly lower than those of workshop workers in the mental health and bodily pain domains, which can be explained by poor working conditions such as lack of adequate furniture in the participants’ homes, resulting in poor posture. Additional factors were lack of specific times for daily meals and for end of working hours, lack of health insurance support, lack of time for rest and leisure, long working hours added to housework hours, and occurrence of a greater number of RSIs, while workshop sewers had access to ergonomic environments, workplace stretching, and predetermined meal breaks.^35^^,^^36^ Similarly, in the study conducted by Costa et al.,^13^ women had poorer QoL because of extra working hours, and, in the aforementioned study of sewers, 95% of the sample was composed of women.

A study conducted by Teodoro et al.^37^ to evaluate ceramic manufacturing workers in the Santa Catarina coal-mining region found that the domains that most influenced the psychophysiological burden for these workers were physical health, psychological health, and environment, the latter being the most unfavorable. These findings corroborate the results of the aforementioned study that evaluated QoL in workers of a medium-sized company in the state of São Paulo that produces school and office supplies.^18^ Such findings may be related to exposure to dust, smoke, machine noise, and smell of dyes and products used for manufacturing these supplies, as workers must take some of these products to an oven at high temperatures. All of these environmental factors may lead to poor health.^13^^,^^36^

In Brazil, QoL studies focus mainly on individuals who are ill or health professionals; however, workers’ QoL in the industrial setting remains to be elucidated.^14^^,^^37^ This shows the importance of further research to address this population and investigate the use of interventional measures in the workers’ lifestyle and health, aiming to contribute to the improvement of workers’ QOL.

## CONCLUSION

Few studies assessing QoL of industrial workers were found. The reviewed studies showed that there is a QoL deficit in these workers, especially in the vitality, physical functioning, general health, environment, and psychological health domains, due to the working conditions to which they are subjected, influencing their daily lives. To advance scientific knowledge about the risk factors that workers are exposed to, further studies are required to explore overall QoL in the workplace.

Workers’ health must be seen from a comprehensive perspective, including a full assessment of individuals in all their biopsychosocial aspects. Scientific evidence about QoL has showed the importance of implementing intervention strategies in the workplace to improve the QoL of these workers.
